# A novel blood-based assay for treatment monitoring of tuberculosis

**DOI:** 10.1186/s13104-021-05663-z

**Published:** 2021-06-30

**Authors:** Alexandra J. Zimmer, Samuel G. Schumacher, Erik Södersten, Anna Mantsoki, Romain Wyss, David H. Persing, Sara Banderby, Linda Strömqvist Meuzelaar, Jacqueline Prieto, Devasena Gnanashanmugam, Purvesh Khatri, Stefano Ongarello, Morten Ruhwald, Claudia M. Denkinger

**Affiliations:** 1 Departments of Medicine and of Epidemiology, Biostatistics & Occupational Health, McGill University, Montreal, Canada; 2 FIND, Chemin des Mines 9, Geneva, 1202 Switzerland; 3 Cepheid AB, Solna, Sweden; 4 Cepheid, Sunnyvale, CA USA; 5 Institute for Immunity, Transplantation and Infection, Stanford University School of Medicine, Stanford, CA 94305 USA; 6 Department of Medicine, Center for Biomedical Informatics Research, Stanford University School of Medicine, Stanford, CA 94305 USA; 7 Division of Tropical Medicine, Center for Infectious Diseases, Heidelberg University Hospital, Heidelberg, Germany

**Keywords:** Tuberculosis, Host transcriptional signature, Treatment monitoring

## Abstract

**Objectives:**

A novel 3-gene host transcriptional signature (*GBP5*, *DUSP3* and *KLF2*) has been validated for tuberculosis (TB) treatment monitoring using laboratory-based RNA sequencing platforms. The signature was recently translated by Cepheid into a prototype cartridge-based test that can be run on the GeneXpert instrument. In this study, we prospectively evaluated the change in the expression of the cartridge-based 3-gene signature following treatment initiation among pulmonary TB patients who were microbiologically cured at the end of treatment.

**Results:**

The 3-gene signature expression level (TB score) changed significantly over time with respect to baseline among 31 pulmonary TB patients. The greatest increase in TB score occurred within the first month of treatment (median fold-increase in TB score: 1.08 [IQR 0.54–1.52]) and plateaued after 4 months of treatment (median TB score: 1.97 [IQR: 1.03–2.33]). The rapid and substantial increase of the TB score in the first month of treatment holds promise for the early identification of patients that respond to TB treatment. The plateau in TB score at 4 months may indicate early clearance of disease and could direct treatment to be shortened. These hypotheses need to be further explored with larger prospective treatment monitoring studies.

## Introduction

Closing the diagnostic gap is a priority in the response to TB. In 2019, approximately one third of the estimated 10 million new tuberculosis (TB) cases were undiagnosed [1]. The underdiagnosis of patients presenting at health facilities must be addressed through the scale up of more accurate, non-sputum-based biomarkers and diagnostics. Treatment monitoring tools are particularly limited as smear and culture-based methods remain the standard, and no test of cure is available.

Treatment monitoring is also important for the development of anti-TB therapeutics in clinical trials. The US Food and Drug Administration (FDA) qualified sputum lipoarabinomannan (LAM) as a pharmacodynamic biomarker for quantitatively measuring the bacterial load in sputum [2]. The TB LAM ELISA kit by Otsuka is used as a tool for evaluating the response to treatment during clinical drug development trials for active pulmonary TB [2, 3]. However, a blood-based test, as described here, is expected to have more impact than such a sputum-based test as the quality of blood should remain more constant along the course of treatment and among patient populations.

Thus, genetic host blood transcriptional signatures are promising biomarkers for active disease diagnosis, predicting progress to active TB disease, and treatment monitoring [4]. Such host blood signatures identified and validated for treatment monitoring using laboratory-based RNA sequencing platforms include a 320-transcript, a 664-transcript, the RISK6 and the RISK11 signatures [5–7]. However, the most widely validated signature is a parsimonious combinatory 3-gene score (TB score) identified by Sweeney et al. (*GBP5*, *DUSP3* and *KLF2*), which was found to be associated with disease severity and normalized after treatment initiation [8]. A prospective study by Warsinske et al. validated the 3-gene signature as a diagnostic and treatment monitoring tool while Francisco et al. demonstrated that two of the three genes (*GBP5* and *KLF2*) could be useful for monitoring treatment response in whole blood [9, 10].

In order to move this 3-gene signature to scale, it must be integrated into a testing platform. Cepheid (Sunnyvale, CA, USA) has recently developed an early-prototype cartridge-assay (“Xpert MTB Host Response” or Xpert-MTB-HR-Prototype) to detect the signature expression levels in whole blood using GeneXpert. The Xpert-MTB-HR-Prototype is the first host-response-based blood gene signature to be integrated into an assay for commercial treatment monitoring.

Södersten et al. first evaluated the diagnostic accuracy of the Xpert-MTB-HR-Prototype as a triage test for TB among people living with HIV. The area under the curve (AUC) for the Xpert-MTB-HR-Prototype was 0.89 (95% CI 0.83, 0.94) against a comprehensive microbiological reference standard [11].

In this exploratory study, we assessed whether the Xpert-MTB-HR-Prototype can detect changes in TB score after the onset of treatment among microbiologically cured patients. Our findings could inform future studies to investigate the potential application of the Xpert-MTB-HR-Prototype as a treatment monitoring tool for the early identification of patients that respond to TB treatment and potentially allow for treatment shortening.

## Main text

### Methods

We obtained a total of 185 PAXgene blood samples from 31 patients all  ≥ 18 years with presumptive pulmonary TB symptoms and a positive smear and/or Xpert result and no history of anti-TB therapy in the 60 days prior. Patients were enrolled at the Phthisiopneumology Institute in Moldova (n = 16), Universidad Peruana Cayetano in Peru (n = 10) and the University of Cape Town in South Africa (n = 5) between November 2015 and April 2017. Ethical approvals for the study were obtained respectively from the Phthisiopneumology Institute Chiril Draganiuc (PPI-NRL, Moldova, 7th April 2016, Ref. CE-19.1), the Faculty of Health Sciences UCT Human Research Ethics Committee (UCT, South Africa, 13th February 2014, Ref. 192/2012), and the Ethics Committee of the Universidad Peruana Cayetano Heredia (UPCH, Peru, 11th April 2013, Ref. 4673–4785). The study was undertaken in accordance with the principles of the Helsinki Declaration. Informed written consent was obtained from patients who agreed to participate. Study participation did not affect the standard of care received by the patients.

No resistance was identified and participants were started on first-line TB treatment after enrollment with 5 (16.1%) completing treatment by 6 months and the remaining 26 (83.9%) by 12 months. Patients were considered cured if treatment was completed by 6 months or 12 months, microbiological testing was negative at 6 or latest at 12 months, and symptoms were improved or resolved.

Patients were assessed at initiation of treatment (month 0) and followed-up at 1, 2, 4, 6, and 12 months. Sputum samples were tested on MGIT liquid culture (Becton Dickinson, Franklin Lakes, USA), solid culture on Löwenstein-Jensen (LJ) medium, and smear microscopy at months 0, 6, and 12. Blood samples were collected at every follow up in PAXgene tubes and stored at − 70 °C at the Foundation for Innovative New Diagnostics (FIND) specimen bank (Zeptometrics, USA). Testing using the Xpert-MTB-HR-Prototype (Cepheid Sunnyvale) was performed from biobanked samples at Cepheid (Solna, Sweden) in 2019 as described previously [11]. One cured patient was missing a 3-gene TB score value at the 12-month follow-up.

The 3-gene TB score was calculated using the algorithm established by Sweeney et al. for reverse transcription-polymerase chain reaction (RT-qPCR), defined as [8]:

3-gene TB score = ((GBP5 + DUSP3) / 2)—KLF2).

### Results

**Fig. 1:**
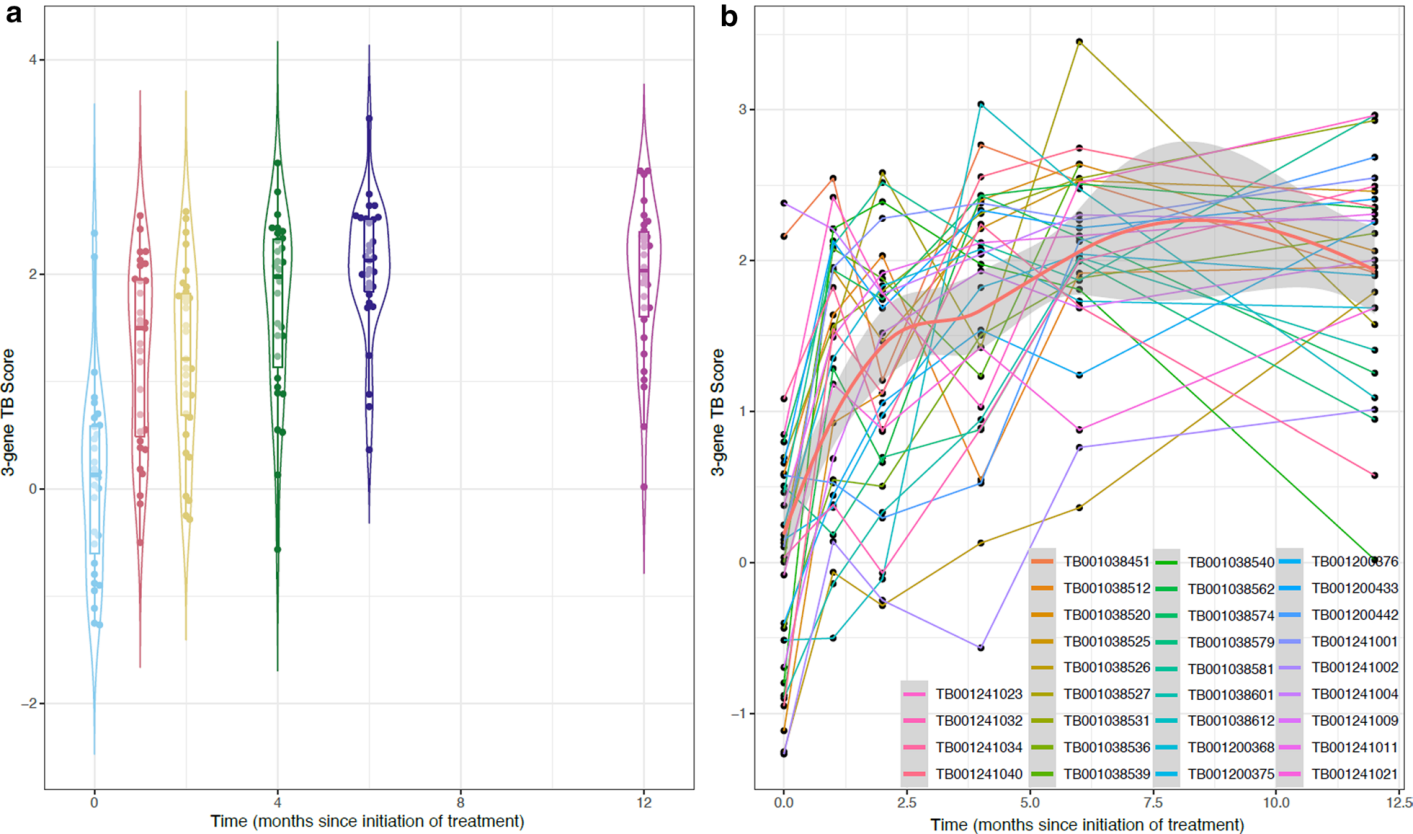
(**a**) TB score at each time point from treatment initiation (month 0) over a 12-month follow-up period among microbiologically cured patients (n = 31). Cycle threshold (Ct) values were used which already exist on the log2-scale, thus the TB score trends appear inverted compared to the data presented by Sweeney et al. [8] (**b**) TB score trajectories for individual patients (n = 31) over a 12-month follow-up period. Grey shading represents the 95% confidence interval

The median age across the 31 participants was 41 years (IQR: 23–57), 19 (61.3%) were male, 27 (87.1%) had a history of BCG vaccination, and 5 (16.1%) were HIV-positive. The median TB scores increased over the follow-up period from 0.13 (IQR: –0.69–0.59) prior to treatment initiation to 2.03 (IQR: 1.58–2.41) by 12 months (Fig. 1a, b). The greatest increase occurred within the first month of treatment initiation with a median increase in TB score of 1.08 (IQR: 0.54–1.52). The median TB score plateaued after 4 months of treatment at 1.97 (IQR: 1.03–2.33). Because the cycle threshold (Ct) values used to compute the TB score already exist on the log2-scale, the TB score trend appears inverted compared to the data presented by Sweeney et al. [8].

### Discussion

In this study, we demonstrate that the Xpert-MTB-HR-Prototype assay detects changes in the 3-gene signature over time with respect to treatment initiation. These findings are in line with published findings on the 3-gene host signature when performed using laboratory-based RNA sequencing platforms [8, 9]. The rapid and substantial increase of the TB score in the first month of treatment holds promise for the early identification of patients that respond to TB treatment. The plateau in TB score at 4 months may indicate early clearance of disease and could direct treatment to be shortened. These hypotheses need to be further explored.

The current 6-month regimen for drug susceptible TB has several challenges including adverse effects and poor adherence. Shorter regimens are needed to overcome these limitations, and the WHO has put forth a Target Regimen Profile that seeks regimens that are at most 4 months in length [12]. A major roadblock in drug development trials is the dependence on long follow up periods for recurrent TB to evaluate treatment efficacy. A more dynamic tool, such as the Xpert-MTB-HR-Prototype, has potential for early up-selection of more promising regimens in shorter and safer trials.

The diagnostic landscape lacks tools that accurately monitor and predict TB treatment outcomes within the early stages of treatment initiation. Such tools would allow patients to receive personalized therapies that ensure relapse-free cure in a timely manner. In this paper, we present preliminary data on the Xpert-MTB-HR-Prototype assay, demonstrating that the change in TB score over time correlates with treatment response in drug susceptible TB patients. Further prospective studies with larger sample sizes are needed to evaluate its diagnostic accuracy and assess its ability to monitor anti-TB treatment response.

## Limitations

There were several limitations to our study. First, the small sample size may have affected the reliability of our findings. Second, our study did not include patients with treatment failure, preventing us from comparing the change in TB score between success and failure cases. Third, we did not recruit a control group (TB negative) in our study and were thus unable to compare the change in TB score against a healthy reference or individuals with other infections or conditions. We note, however, that Sweeney et al. found that the TB scores of patients at recovery (treatment week 28) were not different from those of healthy individuals when measuring the TB score using laboratory-based RNA sequencing platforms [8]. Fourth, we were not able to perform diagnostic accuracy analyses for treatment monitoring. This was due to several limitations in the data collected: 1) a lack of negative control groups (treatment failure or health controls) to compare against; 2) an undefined threshold for the Xpert-MTB-HR-Prototype for treatment monitoring; and 3) a lack of reference standards at interim follow-ups (months 1, 2, and 4) to establish the moment of culture conversion.

## Data Availability

The datasets during and/or analysed during the current study available from the corresponding author on reasonable request.

## Abbreviations

AUC: Area under the curve
Ct: Cycle threshold
HIV: Human immunodeficiency virus
LJ: Löwenstein-Jensen
MGIT: Mycobacterium Growth Indicator Tube
TB: Tuberculosis
WHO: World Health Organization
